# Transparency, bias, and reproducibility across science: a meta-research view

**DOI:** 10.1172/JCI181923

**Published:** 2024-11-15

**Authors:** John P.A. Ioannidis

**Affiliations:** Departments of Medicine, of Epidemiology and Population Health, of Biomedical Data Science, and of Statistics and Meta-Research Innovation Center at Stanford (METRICS), Stanford University, Stanford, California, USA.

## Abstract

N/A

It is a great honor to deliver the AAP Presidential Address. Let me start with disclosures. My main conflict of interest is that I try to be a scientist. This means I am probably biased and often wrong, but hopefully not totally resistant to the possibility of getting corrected. Let me also make some preemptive comments. First and foremost, science is the best thing that can happen to humans, and research should be supported with heightened commitments. You have probably heard this too many times, but it is worth repeating. However, most research done to date has used nonreproducible, nontransparent, and suboptimal research practices. Science is becoming more massive and more complex. Scientific publications (about 200 million already, with 7 million more added each year) are mostly advertisements (“trust me, this research was done”); raw data and experimental materials and algorithms are not usually shared. Moreover, our reward systems in academia and science are aligned with nonreproducible, nontransparent, and suboptimal research practices. Can we do better?

Even though we all use the scientific method, maps of science may visualize many thousands of clusters representing different scientific disciplines (1). The research practices in these many disciplines vary substantially in both expectations and implementation. However, some features are all too common. Notably, the quest for significance is almost ubiquitous. Significance takes many forms, but one form, statistical significance, has become extremely widespread. While originally developed as a helpful discriminating tool for interesting signals, statistical significance gradually became a boring nuisance. Across PubMed, between 1990 and 2015, 96% of the biomedical literature that used P values claimed at least some statistically significant results (2). The good news is that, more recently, a higher rate of “negative” results is tolerated, especially in some study designs such as clinical trials (3). I am not sure whether this is further comfort, but biomedicine is not alone in the significance-chasing frenzy. Actually, empirical data suggest that economics, environmental sciences, and psychology have even higher rates of selective publication reporting bias (4). For example, in economics, roughly 70% of significant results would not have been statistically significant in a bias-free world (5). Also, please note that in medicine we also increasingly see patterns of inverse publication reporting bias (6), in which sometimes “negative” results are preferred. Worrisome examples include studies touting that high-profit drugs, biologics, or vaccines have no significant harms; or noninferiority trials that conclude that a new candidate blockbuster drug that is very expensive is not that much worse than an older, cheaper comparator.

Meta-research entails the study of research practices and the scientific ecosystem at large. Most scientists are trained to focus, to zoom in. In meta-research, scientists mostly zoom out. All scientific disciplines can contribute tools, methods, and empirical data for meta-research. Moreover, science itself is a multifaceted, fascinating object to study.

One may model the scientific ecosystem in broad strokes. For example, 13 equations can create an artificial model universe of science (7). This universe includes diligent, careless, and fraudulent scientists (8, 9). We all hope that the diligent ones are the majority, by far. However, if you ask how many scientists are sloppy or outright frauds, the answer depends on whom you ask and how. Answers to the question, Are you sloppy? rarely receive affirmative answers. However, questions of the type, Are other scientists sloppy? are usually answered with Yes, of course. Fraud is the most difficult to fathom (9). I used to believe that fraud was rare. I suspect my position has become increasingly difficult to defend and wrong nowadays, as I will discuss later.

If we run our modeled universe of science through a number of reward cycles, where scientists get rewarded and create progeny based on what they accomplish (7), the sloppy and fraudulent scientists eventually become the majority. One does not need complex differential equations to understand why. If there are equal rewards for all three categories and no penalties, those who can cut corners, and, even more so, those who get credit with no work at all, just pure fraud, have a competitive evolutionary advantage.

Our reward environment is such that cutting corners and even outright fraud are often tolerated and even incentivized. Some horrible pessimists, who should be canceled and be massively smeared in their Wikipedia pages, dare imagine that one day one of these problematic scientists may even become the president of Stanford or Harvard. Bitter jokes aside, science has become so massive that our traditional ways of correcting the literature are overwhelmed. In most fields, respectable specialty journals have traditionally published a couple of hundred papers each year. Currently, many mega-journals publish more than 2,000 peer-reviewed articles every year; some exceed 10,000 publications annually (10).

Moreover, scientists are attracted to what is hot and incentivized. For example, in 2020 and 2021, 98 of the top-100 most-cited papers across all sciences were on COVID-19 (11). Within 4 years, probably about 2 million scientists published more than half a million papers on COVID-19 (12, 13). These scientists came from all scientific fields. The Science-Metrix classification divides science into 174 fields. Experts in all 174 of these fields published on the topic of COVID-19. The last field to succumb to COVID-19 was automobile engineering, in fall 2020. Most of these scientists ventured way beyond their expertise, in areas in which they lacked even basic skills and understanding. Maybe I should have been excited that everyone overnight became a pontificating epidemiologist, but, well, it was scary. Too much work was also done in haste, cutting corners. Not surprisingly, despite some major scientific successes like vaccines and adaptive randomized trials, most of the peer-reviewed COVID-19 literature was of low quality (14–16) and largely a data fiasco. Worse, science was hijacked not only by outrageous conspiracy theorists, but also by apparently legitimate influencers, journalists, and popular writers. Even if well intentioned, they often promoted and glorified devastating policies such as school closures and aggressive lockdowns (17–19). Many of the things we demanded people do were not just nonevidence-based, they were just weird. I recall walking in the ruins of the Castle of Faneromeni in 2021, a vast desolate expanse on a mountain overlooking the Aegean Sea on the island of Andros. The only person in a two-mile radius was my wife, yet there I was (Figure 1), carrying a double mask, in red and blue colors (perhaps subconsciously hoping for some peaceful, unified bipartisan consensus on the pandemic response). Not surprisingly, trust in science is sadly declining (20).

## Do we need revolution, or would evolution suffice to improve science?

Having published revolutionary manifestos (21), and having created many allies and many enemies as a result, I am currently content to accept evolution even at small but concrete steps. Moreover, I wonder: Should we focus mostly on identifying the problems and hope that their realization will suffice for diligent scientists to fix them or push for aggressive solutions (which may cause more problems, if untested and nonevidence based)?

## Reproducibility has become a buzzword

We all seek reproducibility, but what exactly is reproducibility (22)? We probably all value reproducibility of methods, being able to understand what was done in a study to put the experimental and computational procedures to work again, if needed. But we may disagree about what pains we should take to ensure this. We all wish to have reproducibility of results, additional validation studies that corroborate initial findings. But there is resistance to spending extensive resources purely for replication. Finally, reproducibility of inferences is the most contentious. Even excellent, well-intentioned scientists often reach different conclusions upon examining the same evidence (23).

The typical recipe for research practices involves small-sample-size studies done by solo, siloed investigators and their small teams. To survive in the funding jungle, investigators may cherry-pick nice-looking results. Post hoc narrative building may create works of fiction, as Mitch Lazar has very nicely described in an insightful previous AAP Presidential Address (24). P < 0.05 is enough; there is no registration (“why decrease my data-dredging options?”), no data sharing (“why offer my goldmine to competitors?”), and no replication (despised as a “me too” effort). Small studies suffer power failure, fueling high false-negative and false-positive rates even with limited bias. Power failure is documented in very diverse scientific fields, ranging from neuroscience (25) to economics (26).

An alternative recipe for potential disaster is becoming more common: big data. Extremely large (overpowered) studies, e.g., those fueled by electronic health records, other routinely collected data, and omics platforms, create a firehose of statistically significant results. Still, scientists may build narratives to get funded, so post hoc cherry-picking is still prevalent. Fancier statistical inference tools are often used, but they may be idiosyncratic, lacking consensus. There is no registration of protocols for most of this research. More data sharing occurs, but often without understanding what exactly is being shared. Data users have limited insights into the data generation process.

Small data and big data both have problems, but the worst is stealth research that has no accessible data. The currently discredited Theranos, about which I published the first negative article ten years ago (27) when the company was at its height, was a forerunner of the philosophy that, even in biomedicine, a company should be proud for operating without sharing or publishing their valued data and secrets. While some see Theranos as an isolated failure, the stealth mode is shared by half of the unicorn start-ups in health care fields (28). Moreover, it is becoming particularly prevalent in fields shaping the future of science. For example, in AI, academia and public institutions are currently dwarfs compared with the data availability and computational capacity of tech industries (29). If these companies decline to share, academic and publicly funded research may soon become obsolete. It is equivalent to pursuing microscopy discoveries, with Stanford, Harvard, and NIH having access only to light microscopes, while companies have electron microscopes.

I have absolutely no wish to demonize the industry. In fact, companies are also victims and heroes of the replication crisis. Failed replication in preclinical research was first documented convincingly by big pharma (30). Frustrated by their inability to reproduce research from top academic institutions to put it to work for drug development, the industry published papers documenting the nonreproducibility of academic work. In a landmark Nature paper, in which only six of 53 landmark oncology target projects reassessed by Amgen could be reproduced, the authors concluded that “the failure to win ‘the war on cancer’ has been blamed on many factors . . . But recently a new culprit has emerged: too many basic scientific discoveries . . . are wrong” (31).

Initially, these findings were attacked as biased and nontransparent, since raw data were not shared. However, since then, multiple independent efforts from nonconflicted initiatives have shown similar patterns. Most of the research in our literature is nonreproducible. In the Reproducibility Project: Cancer Biology (32, 33), 193 experiments from top-notch publications were designed, but only 87 could be initiated, and only 50 could be completed. For the rest, the information was insufficient in the published methods and could not even be resurrected in a functional way by communicating with the primary investigators. Most completed experiments showed very different results from what had been published originally. Moreover, the replication process took, on average, 197 weeks — almost four years. You can imagine a PhD student starting their first lab rotation by repeating an experiment from published literature and taking four years to accomplish this introductory task — plus, most of the time failing to make the experiment run.

This uneasy situation leads to “reproducibility wars” with animosity and heated exchanges as reputations are battered. There is also resistance to refutation. Even squarely refuted studies continue to be heavily cited. This paradox has been demonstrated in diverse fields ranging from medicine and epidemiology to psychology (34, 35). Even fully retracted papers may continue to be heavily cited (36).

Several approaches may increase the proportion of true findings. Ten years ago, I published a list of practices that have worked at least in some fields and/or hold high promise (37): conduct of large-scale, collaborative research; adoption of a replication culture; registration (of studies, protocols, analysis codes, datasets, raw data, and results); sharing (of data, protocols, materials, software, and other tools); implementation of reproducibility practices; containment of conflicted sponsors and authors; application of more appropriate statistical methods; standardization of definitions and analyses; establishment of more stringent thresholds for claiming discoveries or ‘‘successes’’; improvement of study design standards; improvements in peer review, reporting, and dissemination of research; and better training of the scientific workforce in methods and statistical literacy.

None of these approaches needs to assume that we have a problem with fraud. It suffices to assume that we have a problem with low efficiency and high waste and that we can honestly improve ourselves. Nevertheless, I have come to revisit my ideas about fraud. For example, the work of John Carlisle is revealing (38). As editor of Anesthesia, a respectable journal, he demanded the raw data from many of the trial papers submitted to his journal. He concluded that 30%–40% of them were “zombie” trials: their results were either entirely messed up or clearly fraudulent. If what happened in Anesthesia applies across the medical literature, I estimated that half a million zombie clinical trials are circulating among us; the editorial was appropriately published on Halloween day (39). Fraud may become more widespread with new AI tools. For example, Wiley recently revealed that when they used a new detection tool, 10%–13% of the 10,000 papers submitted per month in 270 journals were identified as products of paper mills (40). Apparently, fake papers have already massively invaded the scientific literature (41).

Conversely, in other situations, one can fully trust what one reads in a peer-reviewed journal with full transparency. For example, the BMJ and PLoS Medicine have adopted a policy in which all data for clinical trials should be made available to anyone who asks for them. Several years ago, we sent requests to the principal investigators of trials published in these journals, saying that we planned to reanalyze their data. Even though this may have sounded like getting an audit request from the IRS, almost half of the authors sent their datasets. Reassuringly, we obtained results very similar to those in the published record (42).

## There is more good news

More sharing is happening over time across biomedicine, as we have documented in a large-scale evaluation of the entire open-access PubMed Central repository (43). However, there is still large heterogeneity across different scientific subfields in terms of how often they share data or code and how often they register protocols. Moreover, sharing may have plateaued in the last 5 years. New initiatives such as those launched by the NIH (44) and some journals (e.g., BMJ extending the request for data sharing) (45) may further improve the situation, but we should seek empirical evidence to determine whether these initiatives work.

A special mention is due regarding the reproducibility of computational methods. Science is becoming more complex computationally (46). There is an inverse relationship between transparency and complexity (47). More complex computations require extra steps of documentation to open the researchers’ “black boxes.” Transparency, nevertheless, is feasible even in the most complex AI methods (48). Tools are available to make them maximally reproducible. With the advent of large language models, there are further exciting opportunities, but also limitations, challenges, and threats (49). Peer review is also changing rapidly, with multiple new options and major known problems. Even though I am one of the directors of the International Congress on Peer Review and Scientific Publication (50), I have no clue how peer review and scientific publication will look like by 2035. Regardless, it is fascinating to follow the ongoing changes and debates.

No matter how science evolves, we need to reengineer our reward system in ways that incentivize good science of high value and disincentivize poor research practices and waste. We have long focused on productivity, and there is nothing wrong per se with productivity. However, we also need more emphasis on quality, reproducibility, sharing, and translational impact (51). In assessing scientists for hiring, promotion, and tenure we need reliable, responsible indicators (52, 53). Our faculty handbooks are almost always outdated, as we saw in an empirical analysis of the policies of 92 medical faculties worldwide (54).

Scientists’ reputations are based on getting it right, not being right (55). We all make mistakes. The issue is not to deny mistakes and waste but expedite the self-correcting nature of science and improve our efficiency. For those who worry that already six Nobel laureates in medicine or physiology have retracted papers, we can reiterate that science will still be the best thing that can happen to humans, even if all Nobel laureates retract some of their papers eventually. We should remain open to correcting errors, including honest errors, questionable research practices, and outright fraud. Moreover, science engages multiple stakeholders (37). Science affects the lives of billions of people. We need to convince everyone that we do care.

## Figures and Tables

**Figure 1:**
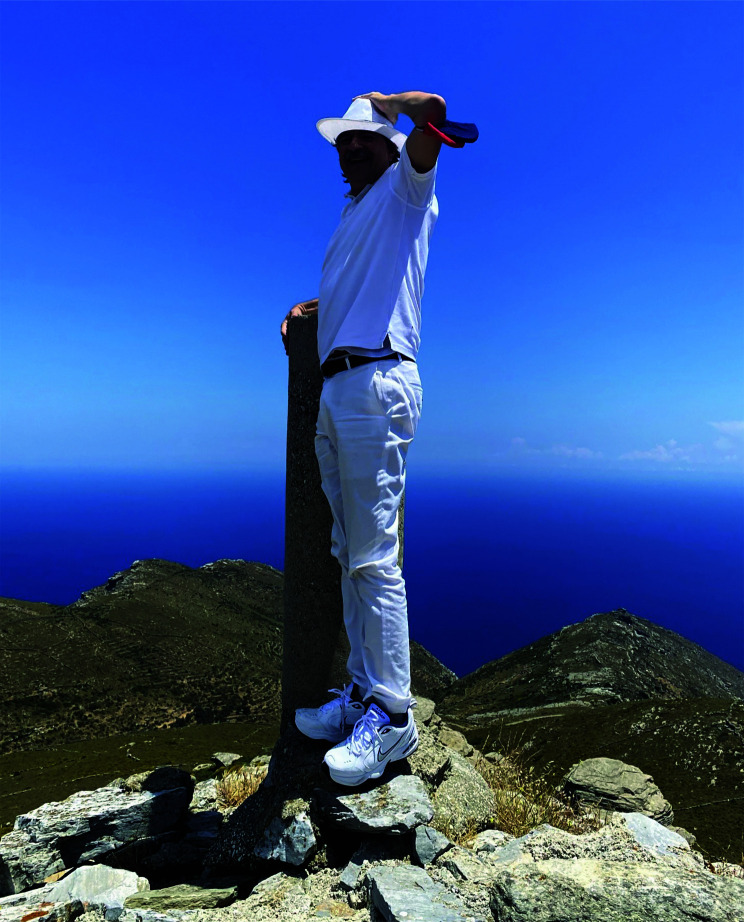
Castle of Faneromeni, Andros, Greece. Offering a bird’s-eye view.

## Version 1 11/15/2024

Electronic publication
